# LY354740, an agonist of glutamatergic metabotropic receptor mGlu_2/3_ increases the cytochrome P450 2D (CYP2D) activity in the frontal cortical area of rat brain

**DOI:** 10.1007/s43440-024-00675-5

**Published:** 2024-11-04

**Authors:** Ewa Bromek, Anna Haduch, Renata Pukło, Władysława A. Daniel

**Affiliations:** Department of Pharmacokinetics and Drug Metabolism, Maj Institute of Pharmacology, Polish Academy of Sciences, Smętna 12, 31-343 Kraków, Poland

**Keywords:** Brain, Frontal cortex, Cytochrome P450 2D (CYP2D), Enzyme activity, MGlu_2/3_ receptor agonist LY354740

## Abstract

**Background:**

Our previous studies indicated that changes in the functioning of the brain glutamatergic system involving the NMDA receptor may affect cytochrome P450 2D (CYP2D) in the brain. Since CYP2D may contribute to the metabolism of neurotransmitters and neurosteroids engaged in the pathology and pharmacology of neuropsychiatric diseases, in the present work we have investigated the effect of compound LY354740, an agonist of glutamatergic metabotropic receptor mGlu_2/3_, on brain and liver CYP2D.

**Methods:**

The activity (high performance liquid chromatography with fluorescence detection) and protein levels (Western blotting) of CYP2D were measured in the microsomes from the liver and different brain areas of male Wistar rats after 5 day-treatment with LY354740 (10 mg/kg ip). The results were analyzed statistically using Student’s t-test.

**Results:**

Among the investigated brain areas, the highest CYP2D activity was found in the cerebellum and brainstem, which exceeded that in the thalamus, cortex, hippocampus and frontal cortex. The mGlu_2/3_ receptor agonist LY354740 administered for five consecutive days significantly increased the protein level and activity of CYP2D in the frontal cortex. Such a tendency was also observed in the other brain areas. LY354740 did not affect the CYP2D activity in the liver.

**Conclusions:**

Repeated administration of the mGlu_2/3_ receptor agonist, the compound LY354740 specifically increases the protein level and activity of CYP2D in the frontal cortex, which may accelerate dopamine synthesis via an alternative CYP2D-mediated route in the mesocortical dopaminergic pathway, and thus may contribute to the beneficial pharmacological effect on negative symptoms of schizophrenia.

**Supplementary Information:**

The online version contains supplementary material available at 10.1007/s43440-024-00675-5.

## Introduction

The cytochrome P450 2D subfamily enzymes (CYP2Ds) metabolize psychotropic drugs, and are engaged in the synthesis of monoaminergic neurotransmitters, such as dopamine and serotonin, and in the hydroxylation of neurosteroids. Thus, changes in the activity of those enzymes may affect the pharmacokinetics and pharmacodynamics of drugs, and, thus, the pharmacological response [1–5]. The expression of CYP2D enzymes involves different transcription factors (hepatocyte nuclear factor HNFα, CCAAT/enhancer binding protein C/EBPα, the farnesoid X receptor-activated transcriptional repressor SHP, peroxisome proliferator-activated receptors PPARs) and miRNAs, the level of which differs between the brain and liver, but the regulation of these enzymes is not yet precisely known [6–8].

In the rat, the CYP2D4 enzyme is mainly expressed in the brain, while the CYP2D1 and CYP2D2 enzymes prevail in the liver [2]. The effects of neuro- and psychotropic drugs on the brain and liver CYP2D enzyme subfamily have recently been observed, while the regulation of brain enzymes was shown to be region-dependent [reviewed by 9]. It seems that different cell types forming brain structure, their innervation, and the presence of pharmacological receptors and transcription factors determine susceptibility to enzyme regulation by xenobiotics. Previous studies indicate that psychotropic drugs regulate brain CYP2Ds mainly at the posttranscriptional level, while modified diets involve also transcriptional mechanisms in enzyme regulation [9–11].

Recent studies examined the influence of the selective antagonist of the GluN2B subunit of the postsynaptic ionotropic N-methyl-d-aspartate (NMDA) receptor, the compound CP-101,606 with antidepressant properties, on the CYP2D expression and activity. They demonstrated increased activity and protein level of CYP2D in the hippocampus after 5 day-treatment, and additional enhancement of the enzyme activity/protein in the cortex and cerebellum after 3-week treatment [12]. Thus, antagonizing the NMDA receptor by CP-101,606 may modify its pharmacological effect by affecting CYP2D-mediated metabolism of endogenous neuroactive substrates in particular brain areas. However, the effects of metabotropic receptors on the brain CYP2D function have not been investigated so far.

The metabotropic group II mGlu_2/3_ receptors are located presynaptically as auto- or heteroreceptors and are negatively coupled to adenylate cyclase activity [13, 14]. Therefore, their stimulation leads to the inhibition of neurotransmitter release [15]. The mGlu_2/3_ receptor antagonists produce an antidepressant effect, while agonists show antipsychotic properties [16]. The compound LY354740 ((1S,2S,5R,6S)-2-aminobicyclo[3.1.0]hexane-2,6-dicarboxylic acid) is a potent, highly selective mGlu_2/3_ receptor agonist. LY354740 shows EC50 at human mGlu_2_ receptor of 5 nM and EC50 at human mGlu_3_ receptor of 24 nM, but no appreciable activity at other human mGlu receptors [17]. The mGlu2/3 receptors are densely expressed in the prefrontal cortex, striatum and hippocampus, i.e. the structures engaged in psychiatric diseases [18]. These brain areas also contain CYP2D protein and display the enzyme activity [19, 20].

The aim of our work was to investigate the effect of repeated treatment with the compound LY354740, an agonist of glutamatergic metabotropic receptor mGlu_2/3_, on the CYP2D expression and activity in the brain. We intended to test a possible contribution of the CYP2D enzyme subfamily to the pharmacological effect of mGlu_2/3_ agonists.

## Materials and methods

### Animals

Wistar Han rats from Charles River Laboratories, Sulzfeld, Germany were used. Male rats weighing 250–300 g (3-month-old) were kept in standard laboratory conditions: temperature 22 ± 2 °C, humidity 55 ± 5%, a 12:12 h light/dark cycle, free access to food and drinking water. All the animals were handled for 5 days before the experiment. Experiments were carried out following the guidelines of the 86/609 EEC Directive and Guide for the Care and Use of Laboratory Animals, and with the permission of the Local Bioethics Committee (the ID number: 307/2017, the date of issue: 7.11.2017) at the Maj Institute of Pharmacology, Polish Academy of Sciences, Kraków.

### Drugs and chemicals

The selective agonist of glutamatergic mGlu_2/3_ receptor, the compound LY354740 (L1045), NADP (N0505), glucose-6-phosphate-dehydrogenase (G7877), glucose-6-phosphate (G7879), bufuralol (UC168) and 1′-hydroxybufuralol (UC169), the mouse monoclonal anti-β-actin primary antibody (A1978) were provided by Sigma (St. Louis, MO, USA). The primary polyclonal anti-rat CYP2D4 anti body (1:1000 dilution) was a gift from the Medical School of Osaka University (Osaka, Japan). cDNA-expressed rat CYP2D4 (Bactosomes) prepared for individual order from Cypex (Dundee, Scotland, UK) was applied. Goat anti-rabbit peroxidase-conjugated secondary antibody (1:2000 dilution) came from Vector Laboratories (PI-1000-1) Burlingame, CA, USA), while goat anti-mouse antibody (115-005-003) (1:2000 dilution) from Jackson ImmunoResearch Laboratories (West Grove, PA, USA). Signal-BoostTMImmunoreaction Enhancer Kit from Millipore (407,207) (Burlington, MA, USA) was used for dilution of the antibodies. The chemiluminescence reagents (LumiGlo kit) (95059-318) were provided by KPL (Gaithersburg, MD, USA).

### Animal treatment and microsome preparation

The selective agonist of mGlu_2/3_ receptor, the compound LY354740 was administered to 10 rats intraperitoneally for 5 days in a pharmacologically active dose of 10 mg/kg [21–23]. The control group (10 rats) was given saline. Two hours after the last dose, the animals were sacrificed by decapitation. Livers and brains were removed and the selected brain structures (the frontal cortical area containing the prefrontal cortex, the cortex, the hippocampus, the thalamus, the brainstem and the cerebellum) were separated according to the atlas of Paxinos and Watson [24]. The isolated tissues were frozen in dry ice, and stored at −80 °C until biochemical analysis. The tissue samples were homogenized and then subjected to differential centrifugation to isolate liver and brain microsomes [25, 26]. The suspended microsomes were stored at −80 °C until analyzed.

### Evaluation of cytochrome P450 2D activity in brain and liver microsomes

The activity of the CYP2D was measured as the rate of CYP2D-mediated bufuralol 1′-hydroxylation [26] in microsomes from selected brain areas or from livers of the control or LY354740-treated rats. Bufuralol was incubated with brain or liver microsomes in a medium containing NADPH generating system, as previously described [12]. The substrate was added to the incubation mixture at a concentration of 5 µM for liver microsomes and 125 µM for brain microsomes (considering a difference in non-specific substrate binding and CYP2D protein levels between the brain and liver). The final incubation volume was 0.4 ml, temperature 37 °C, and reaction time 10 min (liver) or 60 min (brain). The reaction was terminated by adding 30 µl of perchloric acid (70%) and putting the samples in ice. After centrifugation the supernatants were stored at −20 °C until analyzed. Concentrations of the metabolite 1′-hydroxybufuralol were determined by a high-performance liquid chromatography (HPLC) with fluorescence detection at wavelengths of 252 nm (excitation) and 302 nm (emission), as previously described [12, 26].

### Measurement of CYP2D protein level in brain microsomes

The CYP2D protein level was measured in the brain microsomes of control rats or LY354740-treated animals (showing an increase or a tendency to increase the enzyme activity) were estimated by Western immunoblot method. Brain microsome proteins (10 µg of brain microsomal protein) were separated using SDS polyacrylamide gel electrophoresis and relocated onto nitro-cellulose membranes. Next, the proteins were subjected to immunodetection and visualization by means of chemiluminescence as described earlier [12].

The polyclonal rabbit anti-rat CYP2D4 antibody, which reacts with CYP2D4 (main CYP2D enzyme in rat brain) and other CYP2Ds, was applied as primary antibody. The horseradish peroxidase-labeled goat anti-rabbit IgG was employed as a secondary antibody. cDNA-expressed CYP2D4 (2.5 µg) was applied as a standard. The bands’ intensity on a nitrocellulose membrane were measured using the Luminescent Image Analyzer LAS-1000 and quantified by the Image Reader LAS-1000 and Image Gauge 4.0 programs (Fuji Film, Tokyo, Japan). The results of Western immunoblotting were normalized for protein loading based on the β-actin levels. Liver microsomes did not undergo the analysis of CYP2D protein, since the enzyme activity was not changed by the investigated compound.

### Data analysis

The results are presented as the mean ± SEM. Changes in the liver and brain CYP2D activity and brain protein level were statistically evaluated using a two-tailed Student’s t-test. The results were recognized as significant when *p* < 0.05.

## Results and discussion

**Fig. 1:**
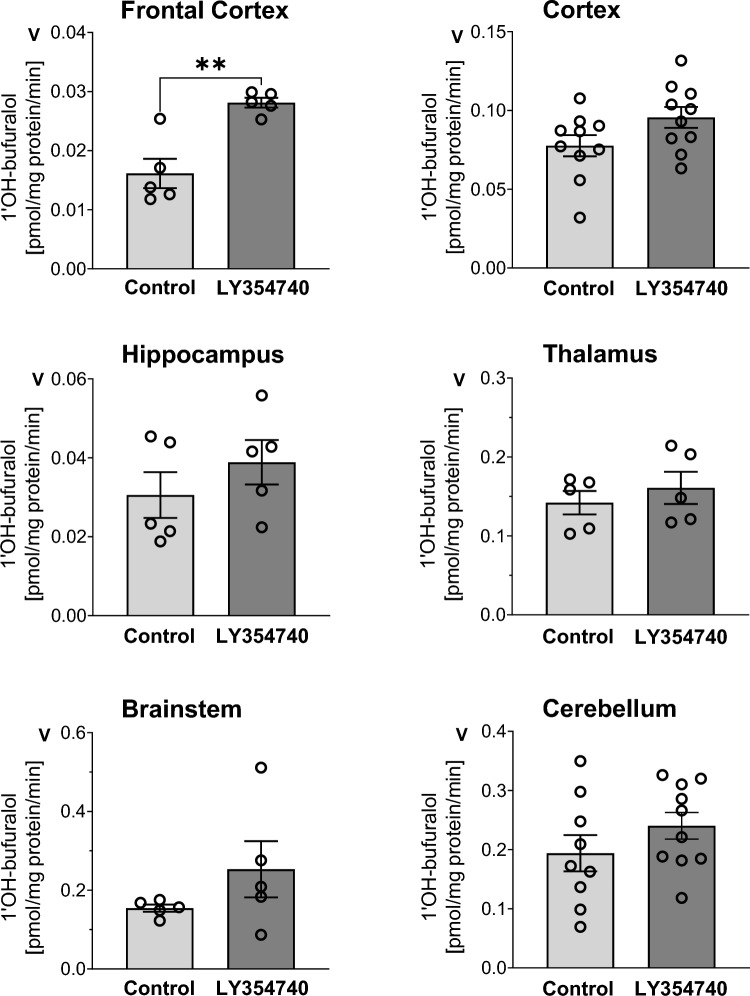
The influence of 5-day treatment with LY354740 (10 mg/kg ip) on the CYP2D activity measured as a rate of bufuralol 1′-hydroxylation in microsomes from the selected brain regions. All values are the mean ± SEM of five samples (each sample consisted of two pooled brain structures from two rats) for most of the studied cerebral structures or of 9–10 samples (from 9–10 rats) for the cortex and cerebellum. The significance of results was calculated using Student’s t-test. Statistical significance is shown as **p < 0.01 vs. control group (*p* = 0.0018;* t*_*8*_ = 4.576)

**Fig. 2:**
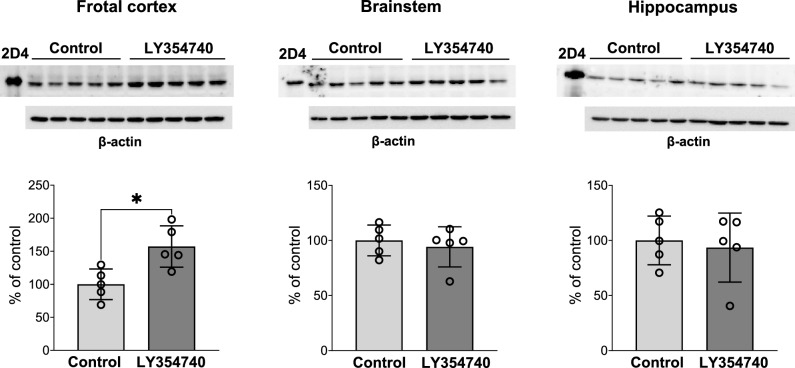
The influence of 5-day treatment with LY354740 (10 mg/kg ip) on the CYP2D protein level in microsomes from the selected brain regions. All values are the mean ± SEM of five samples (each sample consisted of two pooled brain structures from two rats). The representative CYP2D protein bands in Western blot analysis are shown. 10 μg of microsomal protein was subjected to Western blot analysis. cDNA-expressed CYP2D4 (Bactosomes) was used as a positive control. The significance of results was calculated using Student’s t-test. Statistical significance is shown as *p < 0.05 vs. control group *(p* = 0.0111; *t*_*8*_ = 3.283)

Among the investigated brain areas, the highest CYP2D activity was found in the cerebellum and brainstem, exceeding that in the thalamus, cortex, hippocampus and frontal cortex (Fig. 1), which is consistent with our previous studies [20]. The mGlu_2/3_ receptor agonist LY354740 administered for five consecutive days significantly increased the CYP2D protein level *(p* = 0.0111; *t*_*8*_ = 3.283) and activity (*p* = 0.0018; *t*_*8*_ = 4.576) in the frontal cortical area containing the prefrontal cortex (Fig. 1 and 2). A tendency to increase the CYP2D activity was also observed in other investigated structures, but it was not accompanied by a parallel enhancement in the enzyme protein level when measured in the hippocampus or the brain stem.

**Fig. 3:**
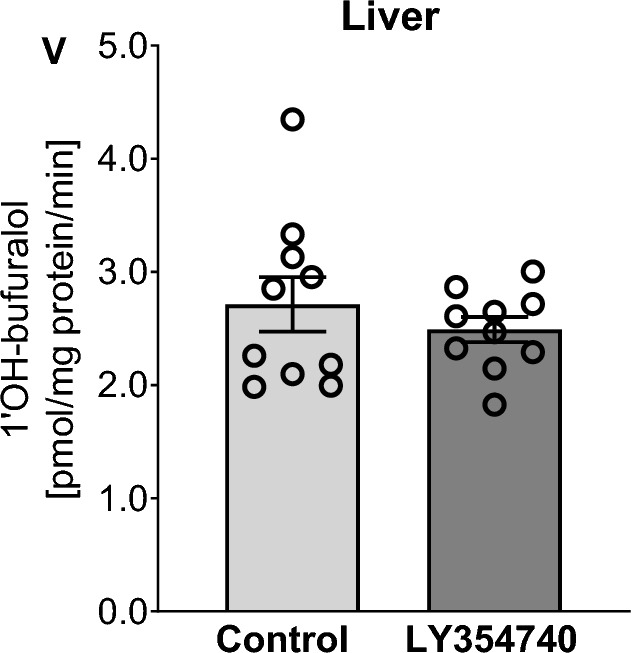
The effect of 5-day treatment with LY354740 (10 mg/kg ip) on the CYP2D activity measured as a rate of bufuralol 1′-hydroxylation in the liver microsomes. All values are the mean ± SEM (n = 10). The results were exasmined statistically using Student’s t-test

The activity of the liver CYP2D subfamily measured as the rate of bufuralol 1′-hydroxylation in liver microsomes was much higher than that in brain microsomes (Fig. 1 and 3), which remains in agreement with previous comparative studies concerning the enzyme level and activity [10, 12, 28]. LY354740 did not affect the activity of the CYP2D enzyme in the liver, which confirms earlier observations of differential, organ- and brain structure-dependent regulation of CYP2D enzyme by neuroactive drugs [reviewed by 5,9,19]. Since the liver CYP2D activity was not changed by the investigated mGlu_2/3_ receptor agonist (Fig. 3), further molecular analysis of liver CYP2D enzyme protein was not carried out.

Interestingly, the prefrontal cortex is a part of the dopaminergic mesocortical pathway engaged in cognitive processes that are impaired in schizophrenia [18]. Dopaminergic projections from mesocortical neurons in the ventral tegmental area (VTA) send their efferents to the medial prefrontal cortex (mPFC), while glutamatergic neurons in the mPFC project to mesocortical dopaminergic neurons in the VTA. This feedback loop regulates dopamine release in the mPFC [27]. Both insufficient and excessive neurotransmitter concentration acting on prefrontal dopaminergic D_1_ receptor may lead to impaired working memory. Therefore, it seems possible that an increase in the CYP2D activity in the PFC, produced by the mGlu_2/3_ receptor agonist LY354740 (as observed in the frontal cortical area containing the prefrontal cortex), leads to an enhancement in the CYP2D-mediated dopamine synthesis, and thus contributes to the regulation of neurotransmitter concentration in the PFC.

Therefore, additional studies are necessary to check whether the observed increase in the activity of CYP2D enzyme leads to the elevation of dopamine concentration in the PFC, and to confirm that the LY354740-evoked increase in the CYP2D activity is due to the mGlu_2/3_ receptor stimulation. Finding out whether other mGlu_2/3_ receptor agonists produce similar effect on CYP2D would be supportive in this respect. Another limitation of the study is that it was performed after 5-day treatment with LY354740. The effect of prolonged administration of different mGlu_2/3_ receptor agonists and antagonists, mimicking their potential clinical application, would be advisable.

The polymorphism of CYP2D enzymes has been implicated in behavior, cognitive processes, learning, and memory in humans and rodents [4]. Prolonged administration of some antidepressants and antipsychotics, investigated earlier, acting via monoaminergic neurotransmitter systems, specifically affected the CYP2D activity in brain areas engaged in depression and schizophrenia, respectively [9]. Recent and present studies suggest that the glutamatergic system modulated via ionotropic or metabotropic receptors may impact the CYP2D activity [12]. This suggests, on the one hand, the involvement of neurotransmitter receptor mechanisms in the CYP2D regulation, and, on the other, a possible contribution of the CYP2D enzyme to the pharmacological effect of psychotropic drugs. Further molecular studies are scheduled to show an association between the type of receptor/intracellular signal and CYP2D regulation in particular brain areas/neuronal cells. However, the implications of the obtained results for human health may be limited, since the results of clinical trials have been mixed, emphasizing the need for understanding mGluR2/3 actions in the primate frontal cortex. It is not excluded that the genetic polymorphism of human *CYP2D6* gene contributes to differential clinical responses to mGluR_2/3_ receptor acting drugs [29]. Moreover, the increased expression and activity of CYP2D in the frontal cortical area may also result in an enhanced metabolism of drugs crossing the blood–brain-barrier and metabolized by this enzyme.

In conclusion, it has been shown for the first time that repeated administration of the mGlu_2/3_ receptor agonist, the compound LY354740, specifically increases the protein level and activity of the CYP2D enzyme in the frontal cortical area containing the prefrontal cortex, which may accelerate dopamine synthesis via the CYP2D-mediated route in the mesocortical dopaminergic pathway, and thus may contribute to the alleviation of negative symptoms of schizophrenia. Further research is necessary to show whether the discovered increase in the CYP2D activity by LY354740 in the PFC area is of pharmacological importance.

## Supplementary Information

Below is the link to the electronic supplementary material.

Supplementary file1 Fig. S1 The original membranes of Western blot experiment. *FCx* the frontal cortical area containing the prefrontal cortex, *Hp* the hippocampus, *Bs* the brainstem (PDF 264 KB)

Supplementary file2 Table S1 Student’s t-test: the values of t, df and p referring to the Figs. 1, 2 and 3 (DOCX 17 KB)

## Data Availability

The datasets are available from the corresponding author upon reasonable request. All immunoblots generated during the study are included in Supplementary Materials.

## Abbreviations

CYP: Cytochrome P450
HPLC: High performance liquid chromatography
NMDA receptor: N-methyl-d-aspartate receptor
PFC: Prefrontal cortex
HNFα: Hepatocyte nuclear factor
C/EBPα: CCAAT/enhancer binding protein
SHP: The farnesoid X receptor-activated transcriptional repressor
PPARs nuclear receptors: Peroxisome proliferator-activated receptors
