# Supplementary figures and images for: LY354740, an agonist of glutamatergic metabotropic receptor mGlu2/3 increases the cytochrome P450 2D (CYP2D) activity in the frontal cortical area of rat brain
Source: Pharmacol Rep. 2024 Nov 4;76(6):1482–8. doi: 10.1007/s43440-024-00675-5 (PMC11582139; doi:10.1007/s43440-024-00675-5)

Western blotting. CYP2D original membranes.

**FCx**

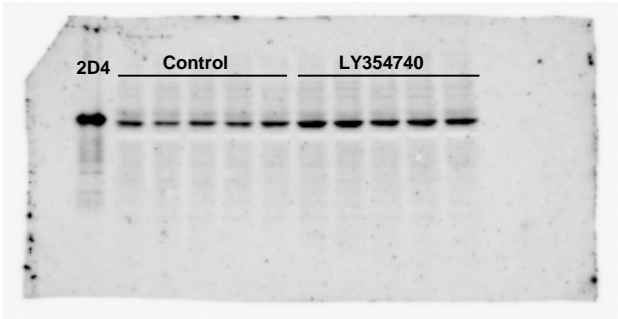

**FCx\_β-actin**

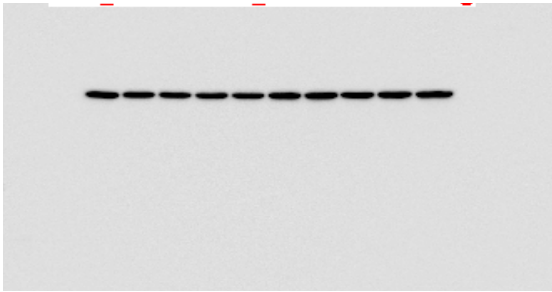

**Bs**

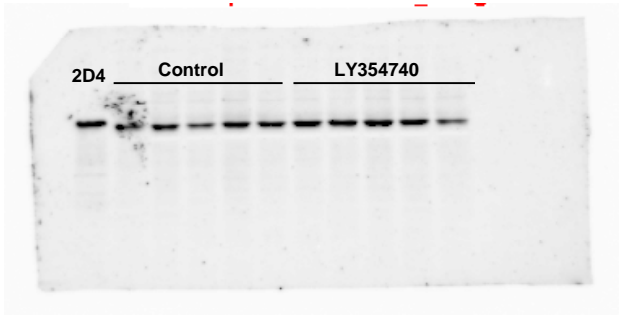

**Bs\_β-actin**

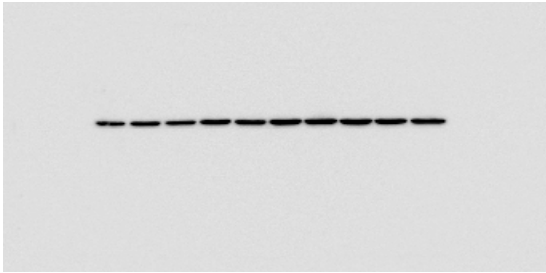

**Hp**

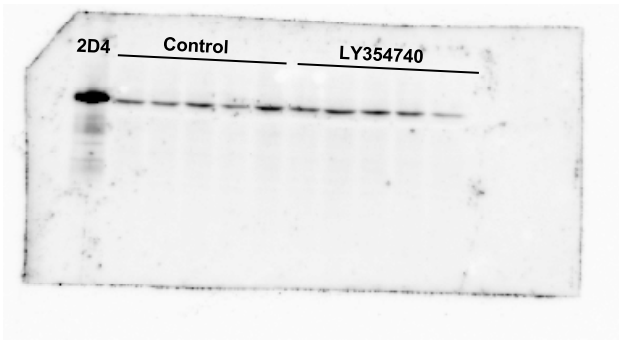

**Hp\_β-actin**

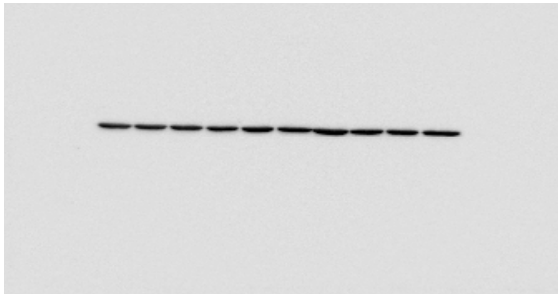

Supplement: Supplementary file 1 — Supplementary file1 Fig. S1 The original membranes of Western blot experiment. FCx the frontal cortical area containing the prefrontal cortex, Hp the hippocampus, Bs the brainstem (PDF 264 KB) [file 43440_2024_675_MOESM1_ESM.pdf]
