# Supplementary material for: LY354740, an agonist of glutamatergic metabotropic receptor mGlu2/3 increases the cytochrome P450 2D (CYP2D) activity in the frontal cortical area of rat brain
Source: Pharmacol Rep. 2024 Nov 4;76(6):1482–8. doi: 10.1007/s43440-024-00675-5 (PMC11582139; doi:10.1007/s43440-024-00675-5)
Supplement: Supplementary file 2 — Supplementary file2 Table S1 Student’s t-test: the values of t, df and p referring to the Figs. 1, 2 and 3 (DOCX 17 KB) [file 43440_2024_675_MOESM2_ESM.docx]

**Table S1.** Student’s t-test: the values of t, df and p referring to the Figs. 1, 2 and 3.

| Fig. 1. The influence of 5-day treatment with LY354740 (10 mg/kg ip) on the CYP2D activity in microsomes from the selected brain regions. Unpaired Student’s t-test, two-tailed | | | |
| --- | --- | --- | --- |
| **Frontal Cortex** | t = 4.576 | df = 8 | p = 0.0018 ** |
| **Cortex** | t = 1.896 | df = 18 | p = 0.0741 |
| **Hippocampus** | t = 1.027 | df = 8 | p = 0.3343 |
| **Thalamus** | t = 0.746 | df = 8 | p = 0.4772 |
| **Brainstem** | t = 1.374 | df = 8 | p = 0.2066 |
| **Cerebellum** | t = 1.238 | df = 17 | p = 0.2327 |
| Fig. 2. The influence of 5-day treatment with LY354740 (10 mg/kg ip) on the CYP2D protein level in microsomes from the selected brain regions. Unpaired Student’s t-test, two-tailed | | | |
| **Frontal Cortex** | t = 3.283 | df = 8 | p = 0.0111* |
| **Brainstem** | t = 0.5714 | df = 8 | p = 0.5834 |
| **Hippocampus** | t = 0.3785 | df = 8 | p = 0.7149 |
| Figure 3. The effect of 5-day treatment with LY354740 (10 mg/kg ip) on the CYP2D activity in the liver microsomes. Unpaired Student’s t-test, two-tailed | | | |
| **Liver** | t = 0.8403 | df = 18 | p = 0.4118 |
